# On-Line pH Measurement Cation Exchange Kinetics of Y^3+^-Exchanged Alginic Acid for Y_2_O_3_ Nanoparticles Synthesis

**DOI:** 10.3390/nano14080696

**Published:** 2024-04-17

**Authors:** Lingyu Liu, Fengchen Zhou, Yuxiang Zhang, Yanhua Sun, Shixing Zhang, Kun Cai, Ruichong Qiu, Yi Lin, Wenjun Fa, Zihua Wang

**Affiliations:** 1 Key Laboratory of Micro-Nano Materials for Energy Storage and Conversion of Henan Province, Institute of Surface Micro and Nano Materials, College of Chemical and Materials Engineering, Xuchang University, Xuchang 461000, China; lly@xcu.edu.cn (L.L.); fczhou@xcu.edu.cn (F.Z.); 18206180781@163.com (Y.Z.); yanhuasun@yeah.net (Y.S.); zhangsx@xcu.edu.cn (S.Z.); caikun2010@gmail.com (K.C.); 15637294563@163.com (R.Q.); 2 School of Chemistry and Chemical Engineering, South China University of Technology, Guangzhou 510640, China; 17665317945@163.com

**Keywords:** on-line pH measurement, alginic acid granules, kinetics study, yttrium oxide nanoparticles

## Abstract

A new sol-gel method that employs cation exchange from an aqueous metal ion solution with H^+^ ions of granulated alginic acid was developed for synthesizing high-purity Y_2_O_3_ nanoparticles. In this study, the cation exchange kinetics of H^+^~Y^3+^ in aqueous solution were analyzed using on-line pH technology and off-line inductively coupled plasma-atomic emission spectrometry (ICP-AES) analysis. Pseudo 2nd-order models were utilized to evaluate the parameters of the kinetics, suggesting that the concentration of H^+^~Y^3+^ involved in the cation exchange reaction was 1:1.733. Further, a comprehensive understanding of the Y-ALG calcination process was developed using thermo-gravimetric analysis, along with results obtained from differential scanning calorimetry (TGA/DSC). A detailed analysis of the XRD Rietveld refinement plots revealed that the crystallite sizes of Y_2_O_3_ nanoparticles were about 4 nm (500 °C) and 15 nm (800 °C), respectively. Differential pulse voltammetry (DPV) was employed to investigate the electrochemical oxidation of catechol. The oxidation peak currents of catechol at Y_2_O_3_ (500 °C)/GCE and Y_2_O_3_ (800 °C)/GCE showed two stages linear function of concentration (2.0~20.0 × 10^−6^ mol/L, 20.0~60.0 × 10^−6^ mol/L). The results indicated that the detection limits were equal to 2.4 × 10^−7^ mol/L (Y_2_O_3_ (500 °C)/GCE) and 7.8 × 10^−7^ mol/L (Y_2_O_3_ (800 °C)/GCE). The study not only provided a method to synthesize metal oxide, but also proposed a promising on-line pH model to study cation exchange kinetics.

## 1. Introduction

The synthesis of metal oxide nanoparticles (e.g., Ce_0.8_Gd_0.2_O_1.90_, NiO-GDC, Ho_2_Zr_2_O_7_, and Ni_x_Fe_y_O_4_) based on sodium alginate gel solutions/granules has been previously developed and reported upon [1,2,3,4]. The alginate gelation is caused by the interaction that is present between the metal cations and the carboxylate groups in a hydrogel solution, with the cation exchange reaction occurring at a rapid rate [5,6]. However, the kinetics of the cation exchange process involving sodium alginate and metal salt solutions has been rarely studied. The relevant study used off-line inductively coupled plasma-atomic emission spectrometry (ICP-AES) to demonstrate a consistent cation exchange kinetics mechanism between Fe^2+^ in an aqueous solution and H^+^ derived from alginic acid [7]. Although ICP-AES technology has certain advantages in the detection of metal ion concentration, the measurement of cation exchange reaction rates is relatively complicated. Therefore, exploring an alternative and efficient method for real-time monitoring of ion concentration changes during cation exchange reactions has been highly imperative.

Alginic acid ([C_6_H_8_O_6_]_n_, H-ALG) is the primary polysaccharide present in brown algae. It is a liner, water-soluble polymer that is comprised of 1 → 4 linked α-L-guluronic acid (G) and β-D-manmuronic acid (M) [8,9]. The cation exchange process between alginic acid and metal salt solution differs from that of sodium alginate (Na^+^) and metal salt solution (M^n+^), and it is mainly based on the H^+^ and M^n+^. Monitoring concentration changes over time using on-line pH technology has been important during the cation exchange reaction process.

Yttrium oxide (Y_2_O_3_), whether pure or doped with other cations, displays interesting chemical and physical properties and, thus, can contribute to various applications such as strengthened steels [10,11], gas sensors [12,13], catalyst [14,15], electrodeposition [16], and transparent ceramics [17,18]. Different methods, such as plasma electrochemistry [19], hydrothermal [15], combustion [10,20], co-precipitation [21], or citrate-assisted sol-gel technique [22], have been reported for synthesizing Y_2_O_3_ nanoparticles. Ensuring rapid, cost-effective, and energy-efficient synthesis of Y_2_O_3_ nanoparticles with ease of scalability has been important from a commercial perspective.

This study primarily aimed to introduce a facile and environmentally-friendly cation exchange process for synthesizing Y_2_O_3_ nanoparticles while making use of granulated alginic acid as a gelation medium. In addition, it provided a new idea to reveal the cation exchange reaction kinetic mechanism. The variation of cation exchange reaction rates between H^+^ and Y^3+^ with an increase in the contact time lengths were studied employing on-line pH and off-line ICP-AES technology. The synthesized samples were characterized by simultaneous thermo-gravimetric analysis, in addition to X-ray diffraction (XRD), transmission electron microscopy (TEM), Brunauer-Emmet-Teller (BET), and differential scanning calorimetry (TGA/DSC). The samples were then employed to modify the glass carbon electrode (GCE) and the electrochemical determination of catechol at the Y_2_O_3_/GCE interface was investigated.

## 2. Materials and Methods

**Figure 1:**
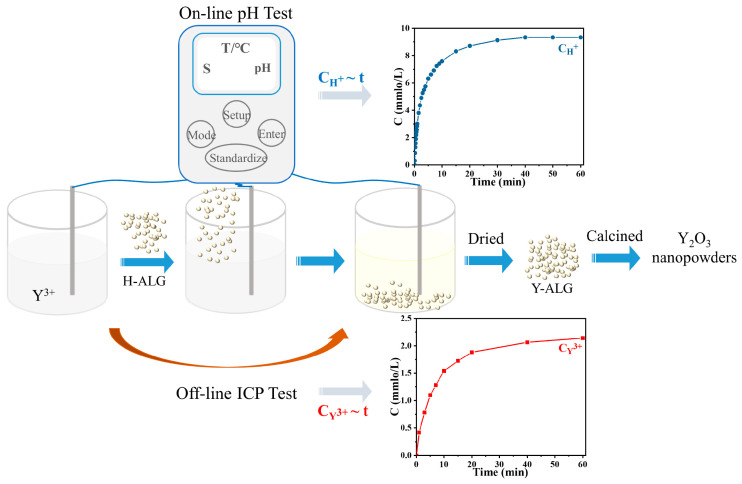
The kinetics of the cation exchange process used for the preparation of Y_2_O_3_ nanoparticles.

Alginic acid (H-ALG), yttrium nitrate hexahydrate (Y(NO_3_)_3_·6H_2_O with 99.99% purity), and catechol were procured from Aladdin Bio-Chem Technology Co., Ltd. (Shanghai, China). Granulated alginic acid was obtained by employing a high shear wet granulator with 200 mL DI water as binger, and employing 1500 r and 250 r as shear rate and agitation rate, respectively. More details on the high shear wet granulation (HSWG) can be found elsewhere [5]. To analyze the kinetics of the cation exchange process between alginic acid and Y^3+^, a 30 g/L Y(NO_3_)_3_·6H_2_O (200 mL) solution was prepared. A pH meter (PB-10, Sartorius, Göttingen, Germany) was immersed in the solution and the initial pH value (t = 0 min) was recorded. This was followed by the addition of 10 g alginic acid granules to the above Y^3+^ solution all at once. Real-time pH changes were recorded at intervals of 0.1 to 60 min while employing slow magnetic stirring to facilitate the cation exchange reaction between H^+^ and Y^3+^. Meanwhile, the Y^3+^ concentrations in the remaining solution after 0, 1, 3, 5, 7, 10, 15, 20, 40, and 60 min of reaction were measured through ICP-AES (PerkinElmer Optima 8300, Waltham, MA, USA). After the 60 min cation exchange reaction, the wet yttrium alginate (Y-ALG) samples were washed with DI water, freeze-dried for 24 h, and underwent calcination in Muffle furnace (500 °C and 800 °C) to obtain Y_2_O_3_ nanoparticles. Furthermore, the effects of different particle sizes and masses of alginic acid, as well as different Y^3+^ concentrations on the cation exchange rate, were also investigated. The experimental details of the process described above are displayed in Figure 1.

The phase evolution of Y-ALG dried samples was analyzed using TGA/DSC (Netzsch STA449F3, Selb, Germany). The experimental procedure involved heating from an ambient temperature to 800 °C though employing a constant ramp rate of 3 °C/min under an air atmosphere and using a sample mass of 6.55 mg. XRD (PANalytical X’Pert3 Powder, Almelo, The Netherlands) was carried out across the range from 2θ = 10–90°, at a 3°/min scan rate. The crystalline sizes and microstructures were determined through TEM (JEOL JEM-2100F, Tokyo, Japan). Rietveld refinement size-strain structural analysis was conducted to determine the average crystalline sizes of the synthesized Y_2_O_3_ samples [4,5]. The specific surface area, pore volume, and pore size of Y_2_O_3_ (500 °C) and Y_2_O_3_ (800 °C) were measured by BET (Microtrac BEL Belsorp-max, Osaka, Japan). The samples were degassed at 125 °C for 12 h in vacuum and measured at 77 K with liquid nitrogen as the adsorbent, using the sample mass of 49.1 mg (Y_2_O_3_, 500 °C) and 48 mg (Y_2_O_3_, 800 °C). The zeta potential and average particle sizes of Y_2_O_3_ (500 °C) and Y_2_O_3_ (800 °C) were analyzed employing Malvern Panalytical Zetasizer Lab with DTS1070 as sample cell.

The Y_2_O_3_ samples synthesized at 500 °C and 800 °C were further dispersed into water using ultrasonication to obtain the Y_2_O_3_ (500 °C) and Y_2_O_3_ (800 °C) suspension, respectively. The suspensions were then employed to alter the GCE. Before its modification, the unmodified GCE underwent polishing with alumina slurry (0.05 μm) to obtain a mirror-like surface, and it was subsequently washed with ultrapure water using ultrasonication. Thereafter, 5 μL of either the Y_2_O_3_ (500 °C) or Y_2_O_3_ (800 °C) suspension was applied onto the surface of the GCE and then dried using an IR lamp to get the Y_2_O_3_ (500 °C)/GCE or Y_2_O_3_ (800 °C)/GCE. The differential pulse voltammetry (DPV) measurements were carried out using a CHI660E Chenhua electrochemical (Shanghai, China) workstation. In the electrochemical measurements, the modified electrode (Y_2_O_3_/GCE) was used as the working electrode, while a Pt wire and a saturated calomel electrode (SCE, saturated KCl) were the counter and reference electrodes, respectively.

## 3. Results and Discussion

### 3.1. The Cation Exchange Reaction Kinetics of H^+^~Y^3+^

**Figure 2:**
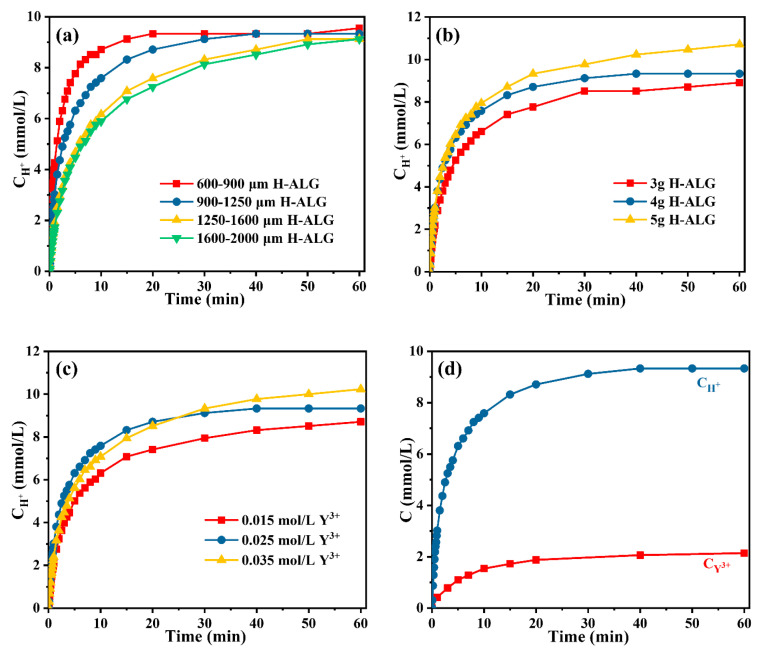
Effects of (**a**) Particle size, (**b**) mass, and (**c**) Y^3+^ concentration on cation exchange reaction rate between Y^3+^ and alginic acid (H^+^) determined through on-line pH measurement; (**d**) The comparison of the concentration of reacted solution between Y^3+^ and H^+^ as the reaction time increased.

The cation exchange reaction kinetics between alginic acid granules (H^+^) and Y^3+^ were investigated via on-line pH technology. The effects of the cation exchange reaction rate of H^+^ and Y^3+^ were studied under different conditions and the results were compared, as shown in Figure 2a–c. Figure 2a depicts the impact of the particle size of alginic acid granules on the cation exchange reaction rate, showing a relatively fast cation exchange. The cation exchange rate was the fastest for the particle size of alginic acid granules in the 600–900 μm range. However, when the alginic acid particles were too small, they tended to dissolve upon contact with the Y^3+^ solution, resulting in the absence of gel formation, thus affecting the final yield of Y_2_O_3_ nanoparticles. When the particle size was further increased to 900–1250 μm, the cation exchange rate became slower. This can be attributed to the higher alginic acid particle density that leads to the slower diffusion rate between H^+^ and Y^3+^ during the reaction process. As the particle size increased from 1250 to 1600 μm, the rate of cation exchange reaction continued to slow down, and it reached its slowest point within the size range of 1600–2000 μm. Based on the above analysis, alginic acid granules with a size range of 900–1250 μm were selected for further experiments investigating the cation exchange reaction.

The effects of the mass of alginic acid granules on the cation exchange rate are displayed in Figure 2b. The observed trend showed an enhancement in the rate as the mass of alginic acid increased from 3 g to 4 g. This can be attributed to a higher release of H^+^ from the alginic acid, therefore, leading to a faster reaction rate. It is important to note that as the mass of alginic acid granules further increased to 5 g, the initial rate of cation exchange reaction between H^+^ and Y^3+^ cations remained consistent with that observed at 4 g. This can be attributed to the initial reaction rate being very fast during the cation exchange reaction and the amount of H^+^ released by alginic acid gradually reaching saturation. In addition, upon reaching a mass of 5 g and increasing the reaction time, the H^+^ concentration in the solution gradually increased, which corresponded with the slowly increasing trend shown in Figure 2b. Therefore, 4 g alginic acid granules were selected for the subsequent experiments based on the above analysis. As shown in Figure 2c, enhancing the concentration of Y^3+^ in the solution (0.015–0.025 mol/L), increased the initial cation exchange reaction rate. This can be due to the more Y^3+^ in the solution, facilitating a more extensive cation exchange reaction with H^+^. Furthermore, when the Y^3+^ concentration was increased to 0.035 mol/L, a slight reduction appeared in the initial rate of the cation exchange reaction between H^+^ and Y^3+^ cations. This can possibly occur due to the initial concentration of Y^3+^ close to saturation as it reacted with H^+^ at the beginning of the reaction. Therefore, the optimal cation exchange reaction conditions of 4 g alginic acid granules (900–1250 μm) and 0.025 mol/L Y^3+^ solution concentration determined with on-line pH technology were selected due to their faster initial reaction rate.

Under the optimal conditions, the cation exchange kinetics curves of C_H_^+^–t, and C_Y_^3+^–t were compared as shown in Figure 2d. The results showed a significantly rapid initial rate of cation exchange, stabilizing to an equilibrium state in 15 min. These results were consistent whether assessed through on-line pH or off-line ICP-AES technology. This can be explained that a rapid cation exchange reaction occurred upon the interaction between the alginic acid granules and the Y^3+^ solution [7]. To further study the cation exchange reaction mechanism, the 1st-order [23], as well as the 2nd-order models [24] were used to effectively fit and analyze the obtained experimental data. The following formula displays the expression of the 1st-order model [23].

(1) $$q_{t}=q_{e}\left[1-e^{-k_{1}t}\right]$$

Here, $q_{t}$ denotes the concentration of ions present in the solution at time t (the reaction time in units of seconds); $q_{e}$ represents the asymptotic value $k_{1}$ which signifies the 1st-order model rate constant.

The 2nd order model [24] is expressed as follows:

(2) $$\frac{t}{q_{t}}=\frac{1}{k_{2}q_{e}^{2}}+\frac{t}{q_{e}}$$

Here, $k_{2}$ denotes the 2nd-order model rate constant.

**Figure 3:**
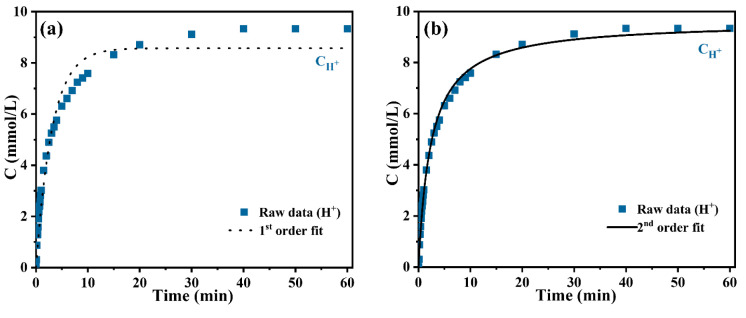

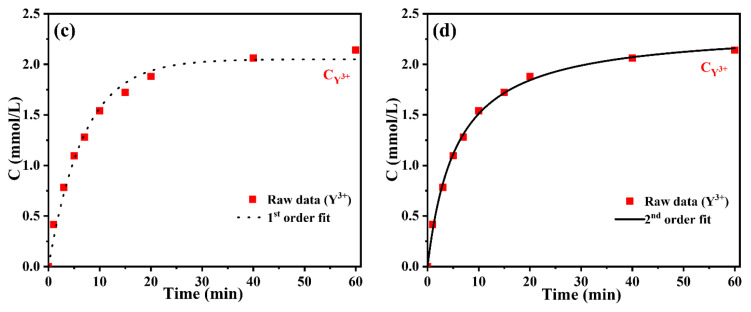
(**a**) 1st-order model versus (**b**) 2nd-order model fit for the H^+^ cation exchange between the aqueous phase and alginic acid granules as a function time through on-line pH. (**c**) 1st-order model versus (**d**) 2nd-order model fit for the Y^3+^ cation exchange between the aqueous phase and alginic acid granules as a function time through ICP-AES.

Figure 3a,b displays the fitting results obtained from employing the 1st-order and the 2nd-order models, respectively, indicating the change in H^+^ concentration over time in the aqueous phase, as measured using the on-line pH method. The results showed a significant fit while employing the 2nd-order kinetic model in comparison to the 1st-order model. The 1st order model accurately fitted the experimental findings in the initial fast cation exchange stage, particularly for the reaction times of lower than 5 min. The 1st-order model fitted well for the initial phase (t < 5 min); however, deviations occurred in case of t > 5 min. On the other hand, the 2nd-order model provided a superior fit for the entire experimental results. The fitting parameters of C_H_^+^–t are displayed in Table 1. The r^2^ value determined from the 2nd-order model was 0.9963, exceeding the value of 0.9656 determined from the 1st-order model. The SD error value for the 2nd-order model (3.48%) was smaller than that of the 1st-order model (32.39%). In addition, Figure 3c,d show the 1st-order and the 2nd-order models fitting results of Y^3+^ concentration change as a function of time using ICP-AES technology. Similar to the fitting results of C_H_^+^–t, the data fitted superior using the 2nd-order kinetic model in comparison to the 1st-order model. The r^2^ value for the 2nd-order model (0.9981) exceeded that of the 1st-order model (0.9898) as shown in Table 1. Compared to the SD error value for the 2nd-order model (0.11%), the SD error value for 1st-order model was higher (0.59%). Considering the analysis of the fitting results, the 2nd-order kinetic model appears to be more applicable for describing this cation exchange reaction process. The rate constant k_2_ for C_Y_^3+^–t was about 1.733 times higher than that for C_H_^+^–t, suggesting that the concentrations of H^+^ and Y^3+^ involved in the cation exchange reaction did not adhere to the theoretical stoichiometric ratio. These findings may provide a theoretical basis for the synthesis of other bimetallic oxide nanoparticles such as yttrium oxide stabilized zirconia.

### 3.2. TGA/DSC of Y-ALG

**Figure 4:**
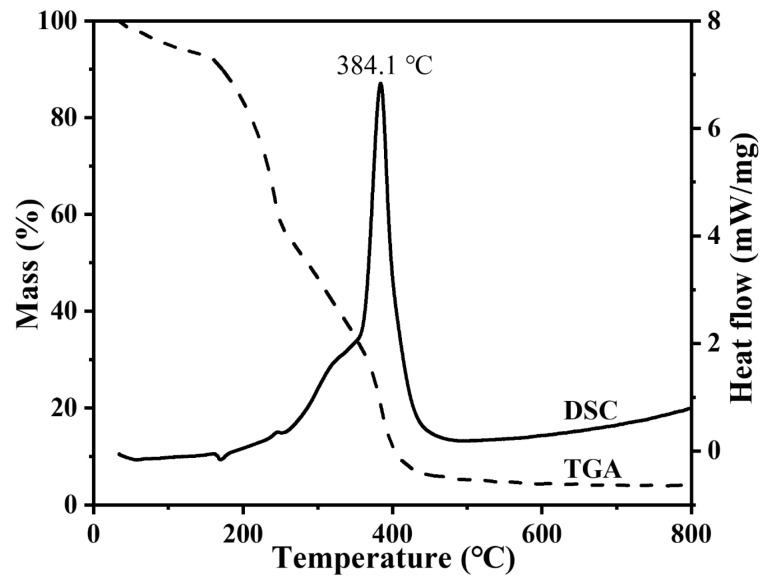
Thermal analysis of Y-ALG from ambient temperature to 800 °C.

To investigate the phase evolution leading to the Y_2_O_3_ nanoparticles synthesis, a simultaneous TGA/DSC analysis was performed employing dried cation exchange Y-ALG granules (after 60 min reaction), as shown in Figure 4. The experiment utilized 6.55 mg of Y-ALG. Three decomposition processes appeared within the temperature ranges of RT 200 °C, 200–300 °C, and 300–450 °C. An endothermic decomposition peak appeared around ~180 °C in the TGA profile, corresponding to an almost 10 wt.% weight loss, which can be attributed to the water evaporation process that occurs during the heat treatment [4,5]. When the temperature was increased further to 300 °C, a weight loss of about 40 wt.% was seen in the TGA profile, corresponding to a small exothermic peak (260 °C) in the DSC profile. Moreover, a sharp exothermic peak appeared in this profile at a peak temperature value of 384.1 °C (see Figure 4). The corresponding loss in weight was about 45 wt.% of the TGA curve between 300 °C and 450 °C. This significant loss could be attributed to the autocombustion reactions of α-L-guluronic acid (G) and β-D-mannuronic acid (M) in the Y-ALG structure. The release of a significant amount of exothermic energy potentially led to the Y_2_O_3_ nanoparticles synthesis [4,5]. No additional weight losses appeared at temperatures exceeding 450 °C, suggesting the completion of the decomposition process (Figure 4).

### 3.3. Characterization of Y_2_O_3_

**Figure 5:**
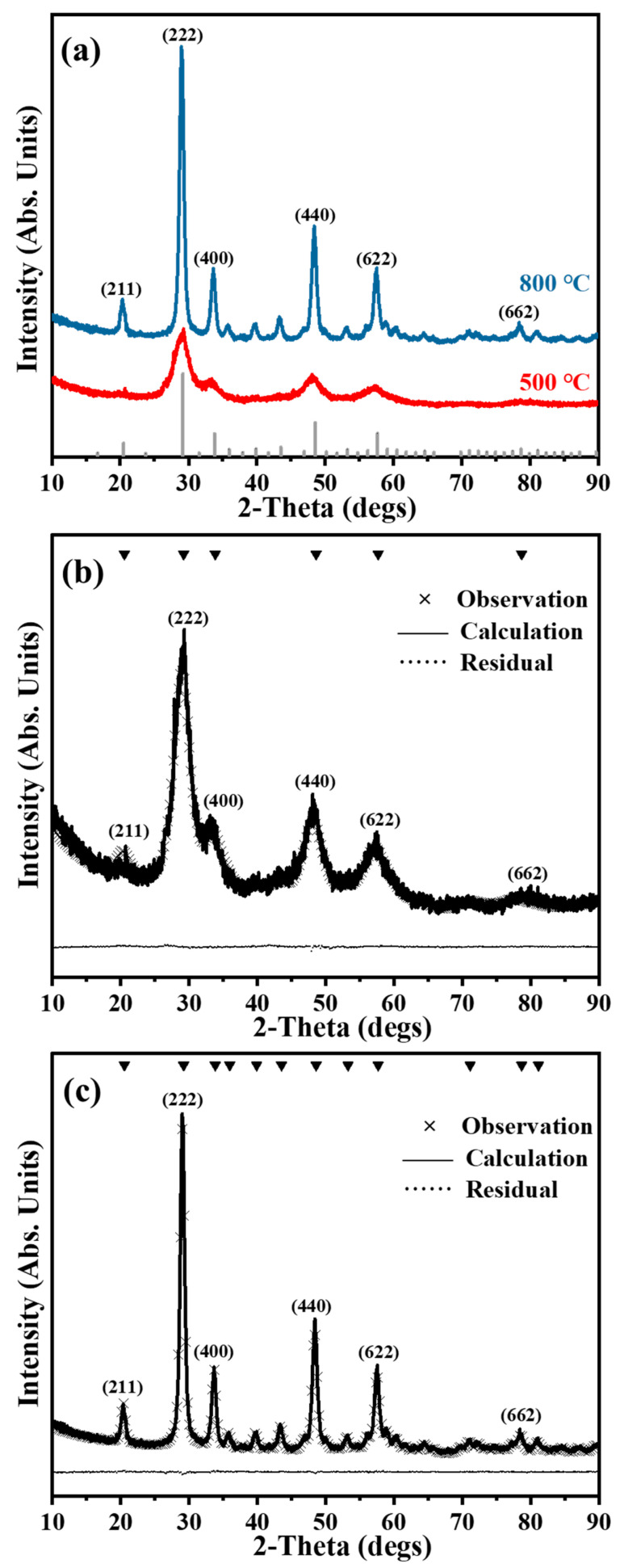
(**a**) The obtained XRD pattern for Y_2_O_3_ calcined at 500 °C and 800 °C. Rietveld structural refinement analysis for Y_2_O_3_ at (**b**) 500 °C and (**c**) 800 °C. Several raw data points were excluded to provide clarity. Indexed by ICSD 86814 as Y_2_O_3,_ as displayed at the top of the peaks (Triangular shape).

The Y_2_O_3_ nanoparticles produced the following calcination between 500 °C and 800 °C for 2 h and were analyzed for their structure employing XRD analysis (Figure 5). The observed XRD patterns showed single-phase cubic structures for all samples. Each peak within the XRD patterns accurately corresponded to the reference pattern (ICSD 86814), as indicated at the top of each peak in Figure 5a. Further, with the increase in calcination temperature, the peaks showed greater sharpness and were narrower because of the increasing crystallite size. Importantly, the XRD patterns acquired at 500 °C were less distinct with much wider peaks than to those observed at 800 °C. Missing peaks were also observed, which could be attributed to the low calcination temperature resulting in the formation of small-sized nanoparticles at 500 °C [4]. Rietveld refinement analysis was used to analyze structural features further, as depicted in Figure 5b,c. Negligible residual differences (nearly a straight line) were observed between the simulated patterns and the experimental measurements. The value of the lattice parameter (10.6101 Å) acquired at 800 °C by Rietveld refinement analysis corresponded to the reference data (10.5961 Å) (Table 2). The average crystallite sizes of the obtained Y_2_O_3_ nanoparticles varied between roughly 4 nm and 15 nm.

**Figure 6:**
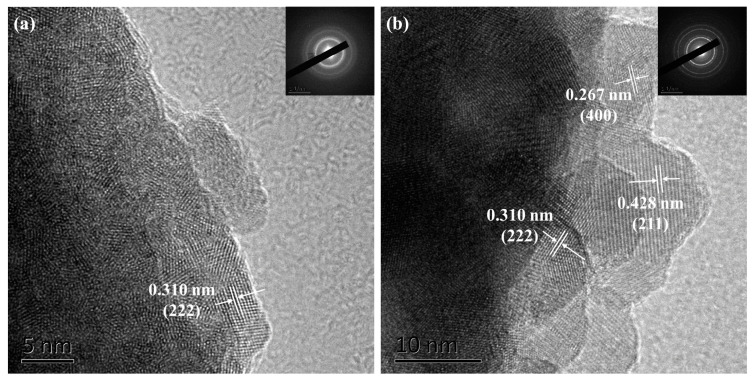
The acquired TEM images for the Y_2_O_3_ nanoparticles, calcined for 2 h at various temperatures: (**a**) 500 °C and (**b**) 800 °C. The insets represent the respective SAED patterns.

Figure 6 illustrates the range of morphologies and crystallite sizes identified via TEM analysis of the Y_2_O_3_ samples after being calcined at 500 and 800 °C for about 2 h. The crystallite sizes range from 4 nm (500 °C) to 15 nm (800 °C). Moreover, the d-spacing values derived from the selected electron area diffraction (SAED) patterns corroborated well with the findings of the XRD Rietveld refinement as detailed in Table 3. The XRD and TEM results with respect to the (111), (220), and (311) crystal planes were quite consistent and agreed well with each other. Errors were more significant in the SAED pattern analysis in comparison to the XRD Rietveld refinement findings because of manual errors that were introduced during analysis of the TEM images, as seen in Figure 6 and Table 3 [4].

**Figure 7:**
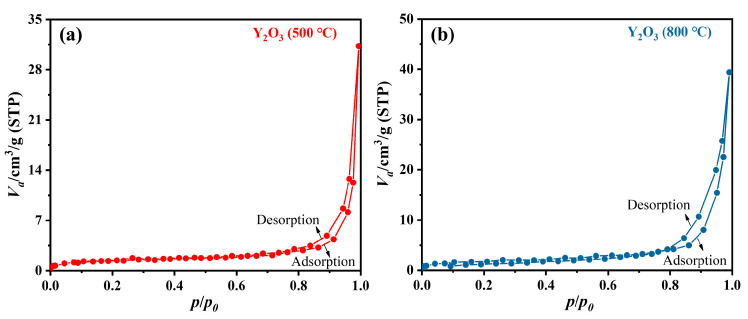
N_2_ absorption-desorption isotherms at 77 K of (**a**) Y_2_O_3_ (500 °C) and (**b**) Y_2_O_3_ (800 °C).

**Figure 8:**
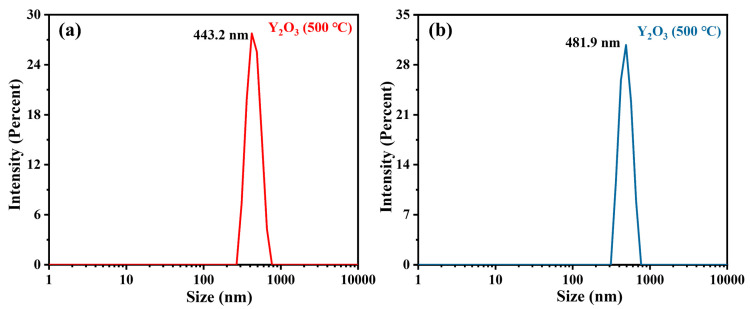
The average particle sizes of (**a**) Y_2_O_3_ (500 °C) and (**b**) Y_2_O_3_ (800 °C).

BET was employed to analyze the specific surface area, pore volume, and pore size of Y_2_O_3_ (500 °C) and Y_2_O_3_ (800 °C). The N_2_ absorption-desorption isotherms at 77 K were shown in Figure 7, indicating the low specific surface areas and the possibility of interparticle pores [25]. Correspondingly, the specific surface areas obtained by linear fitting were 5.1126 m^2^/g (Y_2_O_3_, 500 °C) and 6.2556 m^2^/g (Y_2_O_3_, 500 °C). The pore volume and interparticle pore sizes were calculated by the non-local density functional theory (NLDFT) method [26]. For Y_2_O_3_ (500 °C), the pore volume and interparticle pore sizes were 0.03604 cm^3^/g and 30.454 nm. While, relative higher pore volume (0.05125 cm^3^/g) and lower interparticle pore sizes (26.384 nm) were calculated for Y_2_O_3_ (800 °C). To better understanding of the nanoparticle performances, the zeta potential and average particle sizes of Y_2_O_3_ (500 °C) and Y_2_O_3_ (800 °C) were measured. As shown in Figure 8, the average particle size of Y_2_O_3_ (800 °C, 481.9 nm) were higher than that of Y_2_O_3_ (500 °C, 443.2 nm), corresponding the XRD and TEM results. The zeta potential of Y_2_O_3_ (500 °C) and Y_2_O_3_ (800 °C) were -17.51 mv and -16.12 mv, suggesting the better dispersion of Y_2_O_3_ (500 °C) and stronger electrostatic interaction between Y_2_O_3_ (500 °C) and catechol [27].

### 3.4. Electrochemical Determation of Catechol at the Y_2_O_3_/GCE Electrode

**Figure 9:**
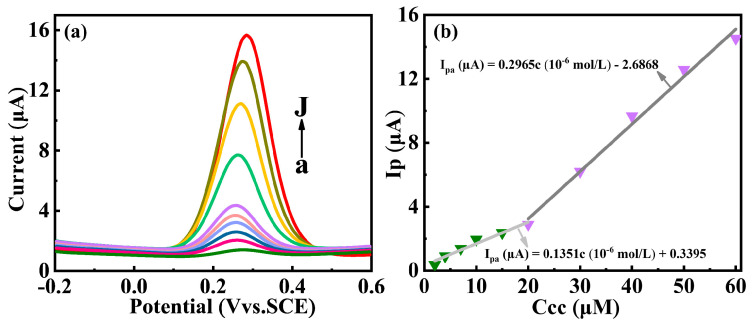

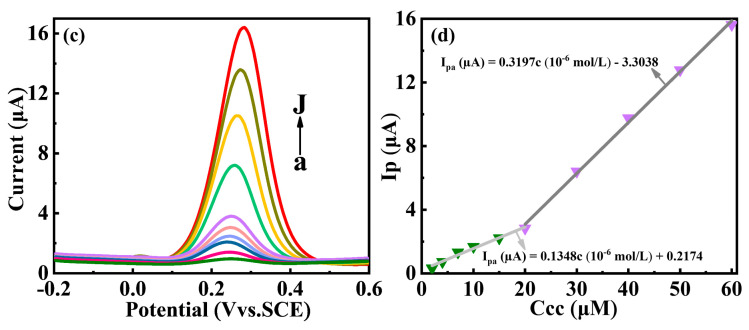
DPV curves of the (**a**) Y_2_O_3_ (500 °C)/GCE and (**c**) Y_2_O_3_ (800 °C)/GCE in 2, 4, 7, 10, 15, 20, 30, 40, 50, and 60 × 10^−6^ mol/L catechol solutions (from a to j), respectively. The linear relationship between the concentration of catechol (CC) and the oxidation peak current on (**b**) Y_2_O_3_ (500 °C)/GCE and (**d**) Y_2_O_3_ (800 °C)/GCE.

The analysis of the electrochemical properties of catechol at the Y_2_O_3_/GCE electrode was performed through DPV, as shown in Figure 9. With the increase in catechol concentrations, the anodic peak current showed an obvious increase (Figure 9a). It can be observed that the peak currents of catechol on Y_2_O_3_ (500 °C)/GCE electrode manifested a linear increase as the catechol concentration increased from 2.0 to 20.0 × 10^−6^ mol/L and from 20.0 to 60.0 × 10^−6^ mol/L respectively, as seen in Figure 9b. Similarly, the tendencies of increase in anodic peak current of catechol on Y_2_O_3_ (800 °C)/GCE electrode and the corresponding linear relationship were observed in Figure 9c and 9d, respectively. With a 3-fold increase in the signal-to-noise ratio (S/N = 3), the detection limit reached 2.4 × 10^−7^ mol/L (Y_2_O_3_ (500 °C)/GCE) and 7.8 × 10^−7^ mol/L (Y_2_O_3_ (800 °C)/GCE). The lower detection limit may be attributed to the smaller crystallite size [28,29] and the lower negative zeta potential of Y_2_O_3_ (500 °C) [27].

## 4. Conclusions

In this paper, single cubic phase Y_2_O_3_ nanoparticles have been fabricated using granulated alginic acid by a cation exchange process using aqueous solutions. During the cation exchange reaction process, the effect of different conditions on the reaction rate of H^+^ with Y^3+^ solution was optimized by the on-line pH technique. The following optimal experimental conditions were selected; a particle size range being 900–1250 μm, 4 g of alginic acid granules, and 0.025 mol/L Y^3+^ concentration. The kinetics of cation exchange reaction were studied by employing on-line pH and off-line ICP-AES technique, and they were demonstrated numerically by the 1st and 2nd order models. The higher r^2^ suggests that the 2nd order model more accurately describes the cation exchange process. Importantly, the result suggested the concentration Y^3+^ involved in the cation exchange reaction was about 1.733 times higher than that for H^+^. The TGA/DSC results indicated the thermal oxidation process of Y-ALG was concluded at 450 °C and led to the synthesis of Y_2_O_3_. The powder XRD results indicated that single-phase cubic Y_2_O_3_ was produced following calcination of dried Y-ALG granules at 500 °C for 2 h. The crystallite sizes measured through Rietveld structural refinement based on the size-strain model showed that the crystallite sizes are about 4 nm (500 °C) and 15 nm (800 °C), respectively. A more negative zeta potential indicated that Y_2_O_3_ (500 °C) had better dispersion and stronger electrostatic interaction with catechol. According to the electrochemical measurements, the synthesized modified electrodes exhibited excellent electrocatalytic activity and low detection limit with 2.4 × 10^−7^ mol/L (Y_2_O_3_ (500 °C)/GCE) and 7.8 × 10^−7^ mol/L (Y_2_O_3_ (800 °C)/GCE).

## Figures and Tables

**Table 1:** The calculated parameters of diffusion models.

C–t	1st Order Fit				2nd Order Fit			
	$k_{1}$	$q_{e}$	r^2^	SD	$k_{2}$	$q_{e}$	r^2^	SD
C_H_^+^–t	0.3217	8.5741	0.9656	32.39%	0.04339	9.6124	0.9963	3.48%
C_Y_^3+^–t	0.1445	2.0504	0.9898	0.59%	0.07523	2.3619	0.9981	0.11%

Standard Error was scaled with square root of reduced Chi-Sqr.

**Table 2:** Rietveld refinement analysis for Y_2_O_3_ acquired under various heat treatment conditions.

Diff. Temp. for 2 h	500 °C	800 °C
Lattice parameter (Å)	10.6997	10.6101
Size (nm)	3.67 (2)	15.26 (9)
Micro Strain	0.75 (1)	0.21 (8)
R_wp_ (%)	10.23	7.94
R_exp_ (%)	7.52	7.53
GOF	1.36	1.05

**Table 3:** Comparison of d-spacing values of Y_2_O_3_ nanoparticles acquired through TEM and XRD Rietveld refinement.

(hkl)	Ref. ^a^ d (Å)	TEM d (Å)	XRD d (Å)
(211)	4.33	4.28 (6)	4.37 (2)
(222)	3.06	3.10 (3)	3.08 (2)
(400)	2.65	2.67 (1)	2.66 (4)

^a^ Reference pattern of Y_2_O_3_ (ICSD 86814).

## Data Availability

Data are contained within the article.
